# Gellified Emulsion of Ofloxacin for Transdermal Drug Delivery System

**DOI:** 10.15171/apb.2017.028

**Published:** 2017-06-30

**Authors:** Swati Jagdale, Saylee Pawar

**Affiliations:** MAEER’s Maharashtra Institute of Pharmacy, MIT campus, Kothrud, Pune (MS) 411038, Savitribai Phule Pune University, India.

**Keywords:** Emulgel, Transdermal, Delivery, Ofloxacin, Antimicrobial, Emulsion

## Abstract

***Purpose:*** Ofloxacin is a fluoroquinolone with broad-spectrum antibacterial action, used in treatment of systemic and local infections. Ofloxacin is BCS class II drug having low solubility, high permeability with short half-life. The present work was aimed to design, develop and optimize gellified emulsion of Ofloxacin to provide site targeted drug delivery. Transdermal drug delivery will enhance the bioavailability of the drug giving controlled drug release.

***Methods:*** Transdermal drug delivery system was designed with gelling agent (Carbopol 940 and HPMC K100M), oil phase (oleic acid) and emulsifying agent (Tween 80: Span 80). Effect of concentration of gelling agent on release of drug from transdermal delivery was studied by 3^2^ factorial design. Emulgel was evaluated for physical appearance, pH, drug content, viscosity, spreadability, antimicrobial activity, in- vitro diffusion study and ex-vivo diffusion study.

***Results:*** FE-SEM study of the emulsion batch B5 has revealed formation of emulsion globules of approximately size 6-8 µm with -11.2 mV zeta potential showing good stability for the emulsion. Carbopol 940 had shown greater linear effect on drug release and viscosity of the formulations due to its high degree of gelling. In-vitro diffusion study through egg membrane had shown 88.58±1.82 % drug release for optimized batch F4. Ex-vivo diffusion study through goat skin indicated 76.68 ± 2.52% drug release.

***Conclusion:*** Controlled release Ofloxacin emulgel exhibiting good in-vitro and ex-vivo drug release proving good antimicrobial property was formulated.

## Introduction

Formulations that are applied to the skin or to mucus membrane are referred as transdermal or topical. Drugs administered through the topical route may have both local and systemic effects, depending on where the application is made and on how the formulation is been constructed. Three different functions may be achieved via application of the formulations to human skin.^1^ First function is the desirability to make the active remain on the surface of the skin, E.g. dermal insect repellents, skin infections, and cosmetics for skin decoration. These pharmaceuticals or cosmetics are called epidermal formulations. The second function is for those topical formulations which are designed for dermal penetration of their actives into the deeper regions of the skin such as the viable epidermis and dermis. These are called as endodermal or diadermal formulations. These formulations are not aimed to get absorbed into systemic circulation. Third function aimed to get the systemic action by transdermal application. Local reactions are undesired in this case. When a systemic effect is sought, topical administration can offer many advantages over oral or parenteral administration. The main advantage includes no first pass effect, the risks and inconveniences of parenteral administration are ignored and large variations in the pH in gastric emptying are avoided. Oppositely, if a local effect is sought; many adverse effects associated with an oral administration can be avoided when the topical route is used.^2^

Emulgels are the one which combine gels and emulsions together. Gelling agent in the water phase converts emulsion into an emulgel. Oil-in-water systems are designed to entrap lipophilic drugs whereas water-in-oil systems are used to encapsulate hydrophilic drugs. Emulsions possess certain degree of elegance and get easily washed off. Emulsions have a high ability to penetrate the skin. Emulgels had many advantages as a pleasing appearance, greaseless, high spreadability, easily washable, thixotropic, emollient, nonstaining, longer shelf life, bio-friendly and transparent.^3,4^

Ofloxacin is fluoroquinolone with broad-spectrum antibacterial activity. Ofloxacin is active on both actively dividing as well as dormant bacteria. The mechanism is by inhibition of bacterial DNA gyrase. Ofloxacin has a wide range of antibacterial activity for the treatment of systemic as well as local infection. The half-life of Ofloxacin is 6-7 hrs. Ofloxacin is slightly soluble in water and methanol. It belongs to BCS class II drug with low solubility and high permeability. Present work was aimed to design emulgel which can enhance the bioavailability of drug and give site targeted delivery. The objective was to optimize controlled release emulgel delivery and to investigate the influence of concentration of gelling agents (HPMC K100M and Carbopol 940) on the drug delivery.

## Materials and Methods

Ofloxacin was gift sample from Mercury laboratories Ltd. HPMC K100M was a gift sample from Colorcon Asia Pvt. Ltd. Oleic acid and Span 80 were purchase from Merck specialties private Ltd. Tween 80 and propylene glycol 400 were obtained from Ranbaxy laboratories Ltd. Carbopol 940 was obtained from Analab fine chemicals. All other chemicals were of analytical grade.

### Drug – excipient compatibility study

Ofloxacin and excipient (HPMC K100M, Carbopol 940) were mixed in 1:1 ratio. As per ICH guidelines samples were kept in the stability chamber (Thermo Lab) for 1 month at 40 ± 2°C/75 ± 5% RH. After one month the samples were evaluated by FTIR, UV spectrophotometer and DSC study.^5^

DSC thermogram was recorded using differential scanning calorimeter (DSC-60, Shimadzu Corporation, Japan). Samples were analyzed at a heating rate of 10°C/min under nitrogen atmosphereover a range of 50-300°C.^6^

### Screening of oils, surfactants and co-surfactants for emulsion formation:

For selecting solvents with good solubilizing capacity for Ofloxacin, the saturation solubility of Ofloxacin in various oils such as oleic acid, vegetable oil, light liquid paraffin, olive oil, castor oil, linseed oil; surfactants such as span 80, tween 80, span 20, tween 20 and co-surfactants such as propylene glycol, propylene glycol 400 were determined. Excess drug was added to 5 ml of oil, surfactant and co-surfactant in a rubber capped vial. They were stirred for 24 hrs on magnetic stirrer at 200 rpm and then the suspension was centrifuged at 5000 rpm. Clear supernatant liquid was separated and filter through Whatman filter paper. Solubility of Ofloxacin was determined by UV spectrophotometer at 287 nm.

Different concentrations of oils were evaluated for preparation of emulsion. Oil was selected on basis of solubility of drug. Batch A2 with composition of Oleic acid (2): Water (8): Tween 80 (0.42): Span 80(0.32) was selected for further study based on its globule size (0.2-0.5 µ).

Changing concentration of co-surfactant i.e. propylene glycol 400 from 0.1 to 0.5ml in batch A2, it was found that batch B5 containing 0.1 ml of propylene glycol 400 to A2 batch gave globule size** in** range of 0.2-0.5 µ, therefore it was selected for further study.

### Preparation of gel phase

Five polymers varying their concentrations namely, carbopol 940 (1 to 5%), HPMC K100M (0.5 to 4%), xanthan gum (2 and 3%), guar gum (1 to 3%) and sodium alginate (2 and 3%), were selected for preparation of gel phase. The carbopol gel was prepared by dispersing carbopol 940 and HPMC K100M separately in sufficient amount of warm water (40-50^o^C) with constant stirring at a moderate speed. Both gels were mixed in 1:1 ratio using homogenizer. The dispersion was neutralized with triethanolamine (TEA) to adjust pH in between 6-7. The gel with guar gum and HPMC K100M were prepared by dispersing the required amount of guar gum and HPMC K100M separately in warm water with constant stirring at moderate speed until gel is formed. Both gels were mixed in 1:1 ratio using homogenizer and by neutralizing the dispersion with triethanolamine, pH in between 6-7 was adjusted. Same procedure was followed for different combinations of carbopol 940 + guar gum, guar gum + xanthan gum, sodium alginate + HPMC K100M, HPMC K100M + xanthan gum.

Combination of carbopol 940 and HPMC K100M were selected to design factorial design.

### Preparation of Emulgel

Ofloxacin (0.3%) was dissolved in oleic acid and stirred for 10 min on magnetic stirrer at 400-500 rpm. Span 80 was added in with stirring on magnetic stirrer at 400-500 rpm. The aqueous phase was prepared by taking required quantity of distilled water. Tween 80 and PEG 400 were dissolved in an aqueous phase with stirring on magnetic stirrer at 400-500 rpm. Oil and aqueous phase were heated separately at 70 -80°C. Then the oil phase was mixed with aqueous phase with continuous stirring.

Carbopol gel was prepared by dispersing Carbopol 940 in purified water with constant stirring at a moderate speed. The gel was obtained by neutralizing the dispersion with triethanolamine. pH was adjusted to 5-7. In case of HPMC K100M gel, it was prepared by dispersing HPMC K100M in warm distilled water (40°C). The dispersion was cooled and kept overnight.

The obtained emulsion was mixed with gel in 1:1 ratio to get an emulgel using homogenizer.

### Experimental design

Concentration of the polymers was decided based on results of trial batches. 3^2^ factorial design was applied. Concentration of carbopol 940 and HPMC K100M was indepenant variables and % cumulative drug release at 8 hrs and viscosity was dependent variables. In Table 1a independent variables are listed and batches were prepared according to the experimental design as per Table 1b.

**Table 1 a:** Selection of variables

**Independent variables**	**Variables (levels)**		
	**High(+1)**	**Medium(0)**	**Low (-1)**
**Concentration of Carbopol 940 (%)**	5%	4%	3%
**Concentration of HPMC K100M (%)**	3%	2%	1%

**Table 1 b:** Factorial Design

**Ingredients (%w/w)**	**F1**	**F2**	**F3**	**F4**	**F5**	**F6**	**F7**	**F8**	**F9**
Ofloxacin	0.3	0.3	0.3	0.3	0.3	0.3	0.3	0.3	0.3
Oleic acid	20	20	20	20	20	20	20	20	20
Span 80	3.2	3.2	3.2	3.2	3.2	3.2	3.2	3.2	3.2
Tween 80	4.2	4.2	4.2	4.2	4.2	4.2	4.2	4.2	4.2
PEG 400	1	1	1	1	1	1	1	1	1
Carbopol 940	3	3	3	4	4	4	5	5	5
HPMCK100M	1	2	3	1	2	3	1	2	3
TEA	q. s. adjust pH 6-7								
Water	q.s.								

### Evaluation of emulsion

#### Globule size measurements 

Droplet size measurements of optimized batch of emulsion were carried out by MOTIC microscope, Field Emission-Scanning Electron Microscopy (FEI-NOVO, NANOSEM 450) and Zetasizer.^7^ In FE-SEM, the sample was prepared by drop casting method with dilution of emulsion. 1 ml of emulsion was diluted with 10 ml of distilled water, and then a drop of emulsion was taken in micropipette and placed on aluminum foil in petri dish. Petri dish was dried at room temperature for 24 hrs. Then the sample was placed in sample holder of FE-SEM and images at different parts were captured.^8^

#### Zeta potential measurement

Globule size and Zeta potential of optimized emulsion batch was measured by zetasizer (Malvern zetasizer, 90) with use of disposable sizing cuvette at 25.1°C. 1 ml emulsion sample was diluted with 10 ml water and result was recorded.

#### Dilution test, pH, Drug content, Viscosity and Centrifugation

To know type of emulsion formed, prepared emulsion was diluted with water which was external /continuous phase.^9^ pH of the emulsions was measured by digital pH meter. Drug content was measured by dissolving known quantity of emulsion in methanol and stirring for 4 hrs. Absorbance was measured at 297 nm in UV/VIS spectrophotometer. Viscosity of emulsions was determined by using Brookfield’s viscometer (spindle No.4). The prepared emulsion was centrifuged at ambient temperature and at 5000 rpm for 10 mins to evaluate the system for creaming or phase separation.^10^

#### Evaluation of Emulgel

Emulgels were evaluated by sensory evaluation for clarity, colour, homogeneity, drug content, presence of particles and fibres. pH was determined with a digital pH meter at room temperature. Viscosity was determined by using Brookfield’s viscometer (spindle No.4).

#### Bio-adhesive strength measurement

The bioadhesion measurement was performed using a modified balance method. Two pans of physical balance were removed. Right side pan was replaced with a 100 ml beaker. On left side, a glass slide was hanged. For balancing the assembly, a weight of 20 g was hanged on the left side. Another glass slide was placed below the hanged slide. Portions of egg membrane were attached with both slides. One gram of gel was placed between two egg membrane faces. Little pressure was applied to form bioadhesion bond. Then slowly water was added on right side beaker, till the gel was separated from one face of attached egg membrane. Volume of water added was converted to mass.^11^ The bioadhesive strength (grams) was calculated from equation 1.

Equation 1 $$\text{Bioadhesive strength }=\;\frac{\text{mg}}{\text{A}}$$

Where, m = weight required to detached the slides, A = Area of rat skin attached to slides, g = Acceleration due to gravity (980 cm/s^2^)

#### Spreadability

Spreadability was determined by an apparatus suggested by Mutimer et al. The apparatus was modified and it consists of a wooden block with pulley at one end. A rectangular ground glass plate was fixed on the block. Gel (about 2 g) was placed on the lower plate and was sandwiched between lower and upper glass plate having the same dimensions, provided with the hook. 500 mg weight was placed on the top of the two plates for 5 min to expel air and to get uniform film of gel. Excess of gel was scrapped off. Upper plate was subjected to a pull of 50 g. Time (sec) required by the upper plate to cover a distance of 10 cm was noted. The spreadability was calculated from equation 2. Shorter the time interval better the spreadability.

S = M x L/ T Equation 2

Where, S = Spreadability, M = weight tied to upper slide, L = length of the glass slide, T = time taken for plates to slide the entire length (sec).

#### In-vitro Diffusion Study

#### Cellophane membrane

A modified Franz diffusion cell was used for permeation study. Cellophane membrane was boiled in distilled water for 1 hr. Then it was soaked in phosphate buffer pH 6.8 for 24 hrs before use. Cellophane membrane was placed between the donor and receptor compartment. 1g of gel was transferred to the donor compartment. Entire surface of membrane was in contact with receptor compartment containing 25 ml of phosphate buffer pH 6.8. The cell was agitated on magnetic stirrer at 50 rpm and maintained at 37±1°C. 2 ml were withdrawn at intervals of 15, 30, 60, 120, 180, 240, 300, 360, 420 and 480 min and was replaced with equal volume of fresh phosphate buffer pH 6.8 each time. Samples were evaluated measuring their absorbance at 287 nm.^12^

#### Egg membrane

Procedure as that of *in-vitro* drug release study was followed. Instead of cellophane membrane egg membrane was used. A small hole was made at the bottom of raw egg to remove all its contents. The egg shell was dipped into 0.1 N HCl for 3 hrs to dissolve the egg shell. Obtained egg membrane was washed with distilled water and used for study. For each time used freshly prepared egg membrane.^13^

#### Ex-vivo diffusion study: Goat skin

Male Goat free from any visible sign of disease was selected. The goat dorsal skin was brought from slaughter house. It was stored in a PSS solution at 37±0.5°C. The dorsal hair was removed and skin was washed with distilled water. Dorsal skin of full thickness was excised and adhering subcutaneous fat was removed. Epidermis facing the donor compartment was mounted on the donor compartment. The receptor compartment contains phosphate buffer solution pH 6.8 at 37 ± 0.5°C. 1 g emulgel was spread. Study was carried out in the similar manner as that with cellophane membrane.^14^

#### Release Experiment / Model dependent method

In order to insight of the drug release mechanism from emulgel, drug release data were examined for zero order, first order, and Higuchi’s model, Hixson and Crowell model, Korsmeyer and Peppas model.^15^

#### Flux

PCP Disso V3 software was used to study the average flux of Ofloxacin from the emulgel (F1 to F9 batches) and marketed formulation (TRIBEN-XT, skin cream) through cellophane membrane.^16^

#### Similarity factor

The drug release from the optimized batch (F4) of the formulation was compared to that of the marketed (TRIBEN-XT, skin cream) formulation. Comparison was carried out by determining the similarity factor. The similarity factor was calculated using the BIT-SOFT software*. In-vitro* diffusion study for the marketed formulation was carried out using the same procedure used for the permeation study through cellophane membrane.

#### Microbiological assay 

This technique is used to study bacteriostatic or fungistatic activity of the compound. Microbiological assay was carried out on the strains of *Staphylococcus aureus* and *Escherichia coli* to study the activity of optimized batch and marketed formulation. For present study sabouraud agar medium and ditch plate technique was used (contain 40 g/liter of Dextrose, 15 g/liter of Agar, pH adjusted to approx 5.4±0.2). Concentration 100 µg/ml of optimized batch (F1 and F4), Pure drug and marketed formulation (TRIBEN-XT, skin cream) were prepared. The overnight grown culture of *Staphylococcus aureus* and *Escherichia coli* was inoculated into the sterilized agar media plates. Four Sabouraud agar medium plates were used. In each agar plate 4 wells were prepared and prepared solutions were filled into the wells. As a standard, distilled water was filled. All four agar plates were kept for incubation for 24 hrs. After 24 hrs the plates were observed and zone of inhibition was measured.^17^ Percent inhibition was calculated as per equation 3.

% Inhibition= L2/L1 x 100 Equation 3

Where, L1: Total length of the streaked culture, L2-: Length of inhibition.

#### Stability study

Stability study was carried on optimized batch F4 to assess its stability after storage using triple stability chamber. The emulgel formulation was packed in clean and dry vial and stored under the accelerated condition for period as prescribed by ICH guidelines. Storage conditions for long term stability was 30°C ± 2°C/65% ± 5% RH and for accelerated stability was 40°C ± 2°C/75% ± 5% RH. Samples were withdrawn at 1, 2, 3 months for long term stability while for accelerated stability conditions samples were withdrawn after 3 months. Samples were evaluated for physical appearance (visually inspected for any change in color and appearance), drug content and viscosity.^18^

## Results and Discussion

**Figure 1:**
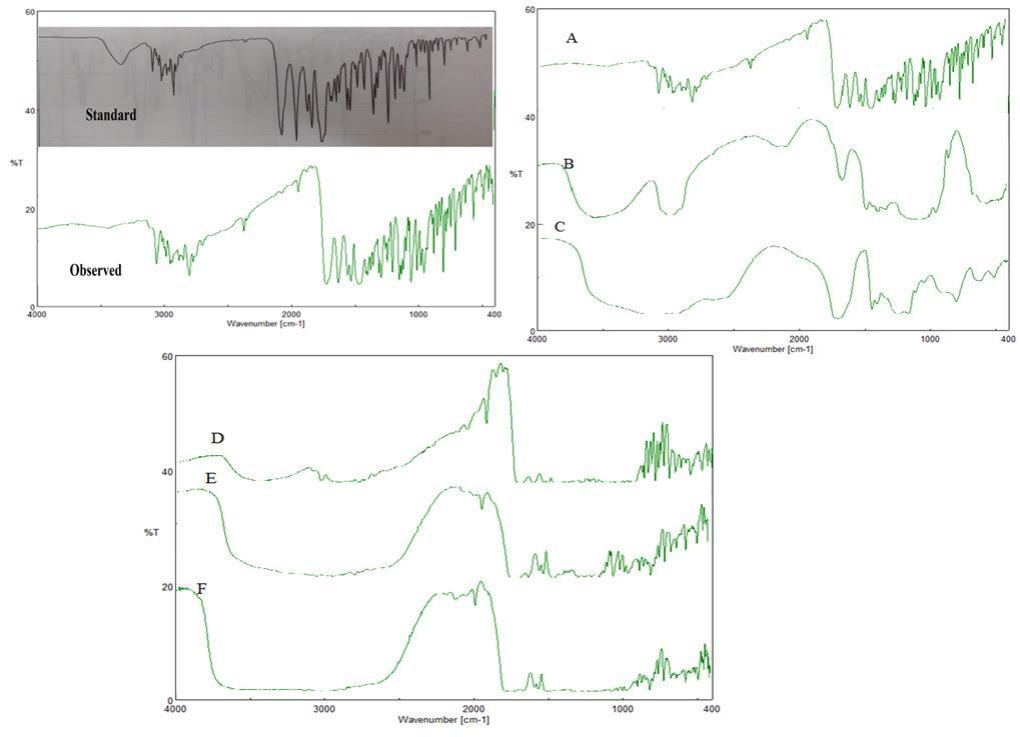
IR Spectrum of Ofloxacin A) Ofloxacin B) HPMC K100M C) Carbopol 940 D) Drug + HPMC K100M E) Drug + Carbopol 940 F) Drug + Carbopol 940 + HPMC K100M

Ofloxacin exhibited characteristic peaks as shown in Figure 1. One prominent characteristic peak was found in between 3050 and 3000 cm^-1^ which was assigned to stretching vibration of OH group and intramolecular hydrogen bonding. Band also suggested NH stretching vibration of the imino-moiety of piperazinyl groups which was less prominent due to intense OH stretching vibration. Peak at 2700 cm^-1^ indicated CH_3_ of methyl group. 1750-1700 cm^-1^ band represented the acidic carbonyl C=O stretching. 1650 to 1600 cm^-1^ peak was assigned to N-H bending vibration of quinolones. Band at 1550 to 1500 cm^-1^ represented CH_2_ of the aromatic ring. Band at 1450-1400 cm^-1^ indicated stretching vibration of CH_2._This had confirmed presence of methylene group in benzoxazine ring. Peak at 1400-1350 cm^-1^ represented bending vibration of hydroxyl group. Band at 1250 to 1200 cm^-1^ suggested the stretching vibration of oxo group. In addition, a strong absorption peak between 1050 and 1000 cm^-1^ was assigned to C-F group. Band at 900-800 cm^-1^ represented the out of plane bending vibration of double bonded ‘enes’ or =CH groups (as per I.P.).

### Drug excipient compatibility study 

After one month accelerated study, major peaks (Figure 1 A) of Ofloxacin were observed at 2936 cm^-1^(CH_2_), 1714 cm^-1^(C=O), 1621 cm^-1^(NH_3_), 1550 cm^-1^(CF), 1459 cm^-1^(CH_3_)and 1086 cm^-1^(CH).

IR peaks (Figure 1B) for HPMC K100M were observed at 2922.59 cm^-1^(C-H), 3420.14 cm^-1^ (N-H), 1058.73 (C-O) cm^-1^.

IR peaks (Figure 1 C) for Carbopol 940 were observed in between 1750 and 1700 cm^-1^ which indicated carbonyl C=O stretching. Band at 1450 to 1400 cm^-1^ indicated C-O / O-H. Band at 1250 to 1200 cm^-1^ was assigned to C-O-C of acrylates. Prominent peak at 1160 cm^-1^ confirmed ethereal cross linking which represented a stretching vibration of C-O-C group. Band between 850 and 800 cm^-1^ indicated out of plane bending of C=CH, i.e., δ=C-H.

The spectrum of drug + HPMC K100M is shown in the Figure 1D which showed peaks at 2936 cm^-1^, 1714 cm^-1^, 1627 cm^-1^, 1550 cm^-1^, 1460 cm^-1^, 2932 cm^-1^, 3422 cm^-1^ and 1060 cm^-1^.

The spectrum of drug + carbopol 940 is as shown in Figure 1E which showed peaks at 2946 cm^-1^, 1720 cm^-1^, 1610 cm^-1^, 1425 cm^-1^, 1230 cm^-1^, 1158 cm^-1^ and 820 cm^-1^.

The spectrum of drug + HPMC K100M + Carbopol 940 is shown in Figure 1 F. The spectrum showed peaks at 2930 cm^-1^, 1708 cm^-1^, 1635 cm^-1^, 1555 cm^-1^, 2940 cm^-1^, 3445 cm^-1^, 1045 cm^-1^, 1430 cm^-1^, 1220 cm^-1^, 1168 cm^-1^ and 825 cm^-1^.

The spectrum of mixtures of drug and excipients has not shown any major change in drug as well as polymer peak. Thus it was concluded that there was no chemical reaction in between drug and proposed excipients.

UV Compatibility study indicated absence of visual changes in the physical mixtures up to 4 weeks, irrespective of storage of samples at ambient conditions. The UV compatibility study indicated absence of any changes in λ max that is 287 nm which concluded that drug was compatible with the proposed excipient.

**Figure 2:**
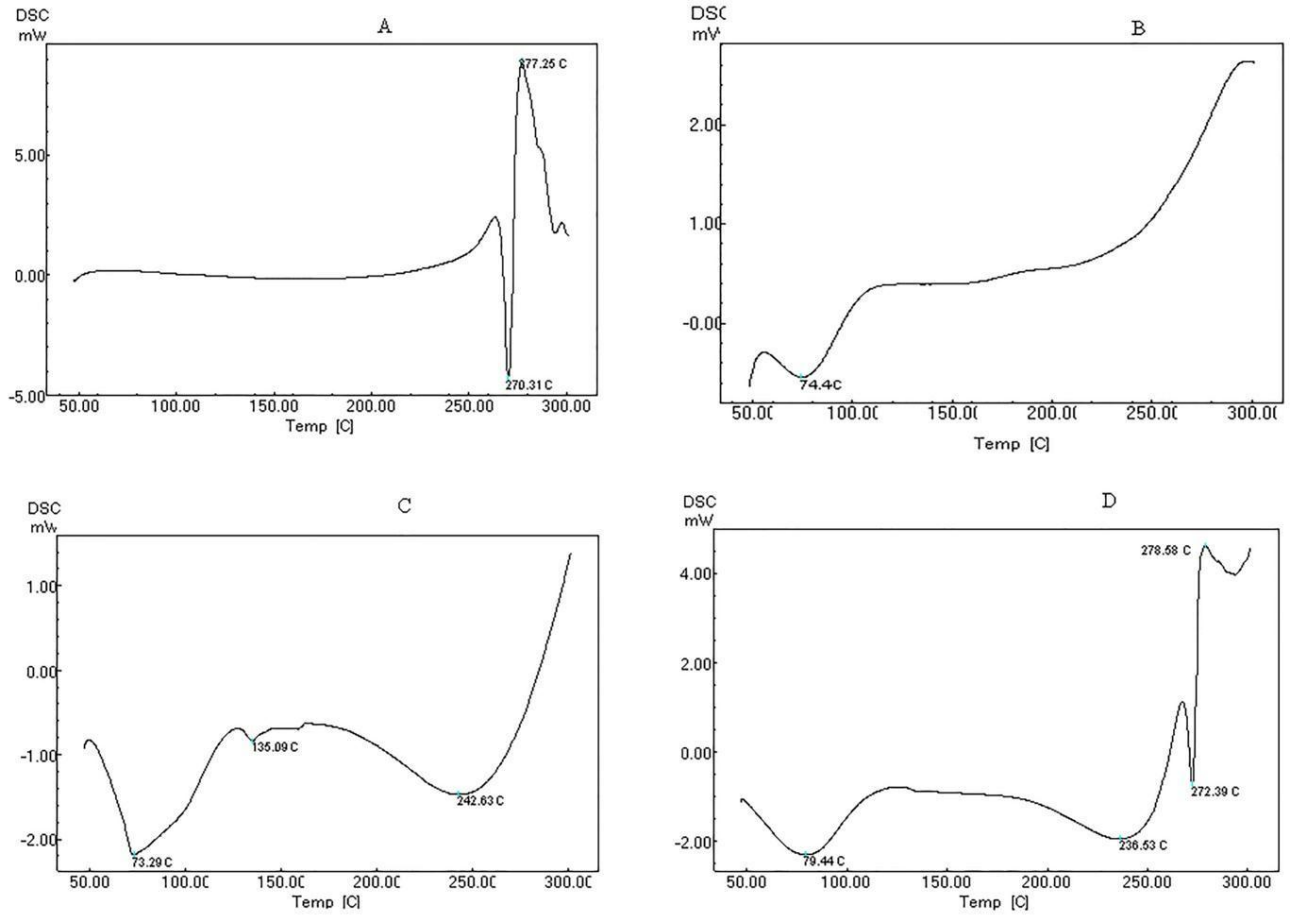
DSC Spectrum for A) Ofloxacin B) HPMCK100M C) Carbopol 940 D) Ofloxacin + HPMCK100M + Carbopol 940

DSC spectrum of pure drug Ofloxacin indicated melting point sharp at 270.31°C (Figure 2A). Sharp peak indicated that the drug Ofloxacin is in crystalline form. HPMC K100M showed melting point at 74.4°C (Figure 2B). Carbopol 940 showed melting point at 242.6°C (Figure 2C). The mixture of plain drug, HPMC K100M and carbopol 940 showed melting point at 272.3°C, 79.4°C and 236.5°C (Figure 2D) respectively. The drug and the excipients had shown no major change in melting points which indicated compatibility.

### Screening of oil surfactant and co-surfactant

Highest solubility of Ofloxacin was obtained in oleic acid (24.88±0.24 mg/ml) amongst oils. Tween 80 (7.31±1.43 mg/ml) and Span 80 (11.88±2.38 mg/ml) amongst surfactants and propylene glycol 400 (3.50±2.10 mg/ml) amongst co-surfactants showed highest solubility. Solubility in water was 4.23±1.10 mg/ml.

From preliminary batches of emulsions, proportion oil and water phase 2:8 (A2 batch) was selected using span 80 and tween 80 as a surfactant. The other batches (A1, A3-A7) were rejected due to its phase separation and consistency of emulsion.

After that, surfactant to co-surfactant ratio 3:1 (B5 batch) was decided by using spans 80 and tween 80 as a surfactant and propylene glycol 400 as a co-surfactant. Oil and water proportion was taken as per batch A2 with span 80 and tween 80 in a beaker and then propylene glycol 400 was added into beaker drop wise with constant stirring. The emulsions were then visually inspected for consistency. The other batches (B1-B4) were rejected due to lack of consistency of the emulsions.

### Selection of gelling agent

From all trial batches of gels, the optimized combination of carbopol 940 and HPMC K100M polymers was selected. Batches of guar gum with HPMC and xanthan gum as well as guar gum with sodium alginate were rejected due to problem in viscosity. Also after storage for 1 week fungus formation was observed in those batches. The batches HPMC with xanthan gum because of its viscosity and spreadability.

### Evaluation of emulsion

All formulation batches were found to be homogenous and milky emulsions.

### Globule size measurement

**Figure 3:**
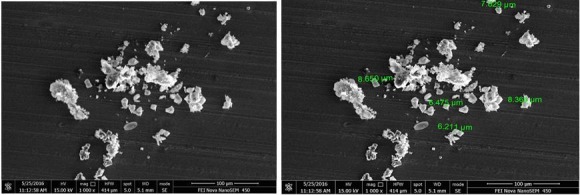
FE-SEM images of optimized batch of emulsion

**Figure 4:**
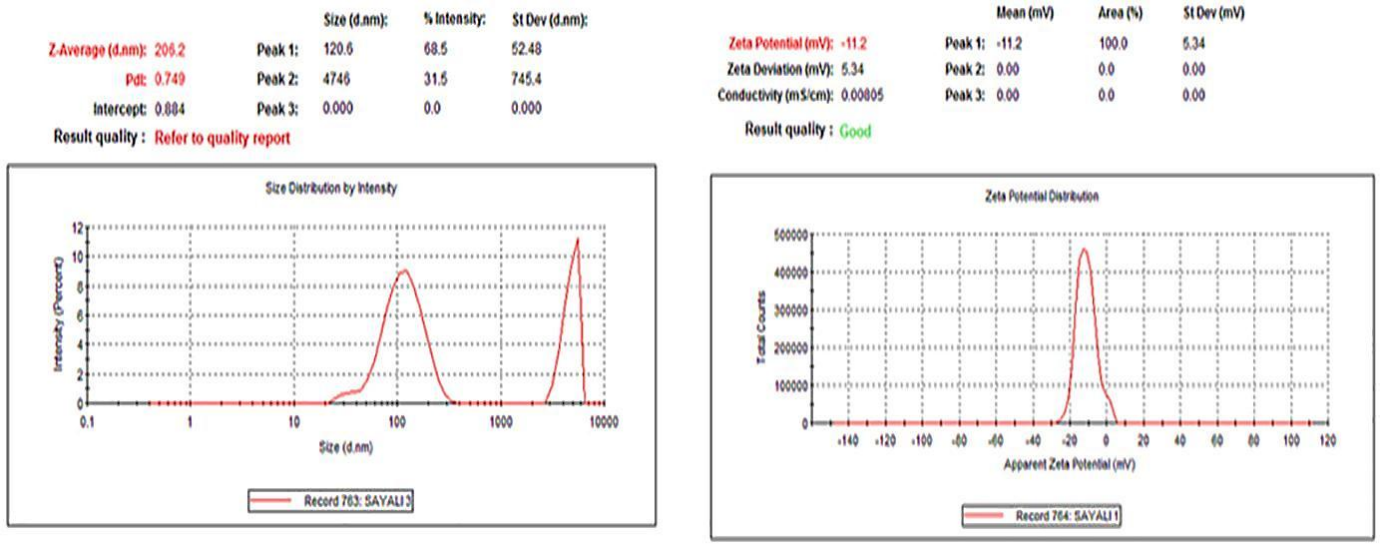
Zeta potential measurement

Motic microscopic image of emulsion had shown spherical shape for globules in size range between 50-200 nm. Field emission-scanning electron microscope(Figure 3) indicated spherical shape for oil globules of optimized emulsion. Size range was in between 6-8 µm. This analysis of emulsion showed conformation of globule size. Globule size was found to be 206.2 nm through zetasizer. PDI is measure of particle homogeneity and it varies from 0.0 to 1.0. If PDI value closer to 0.0, it indicates narrow size distribution of the emulsion. PDI of optimized emulsion was found to be 0.749; hence it indicates prepared emulsion is monodisperse which remains stable and not converted to micro-emulsion. Figure 4 indicates globule size and PDI of emulsion.

### Zeta potential measurement

The general dividing line between stable and unstable emulsion was taken at either +30 or -30 mV. Particles with zeta potential value more positive than +30 mV or more negative than -30 mV are normally considered as stable. Zeta potential of optimized batch was found to be -11.2mV which indicated good stability of the emulsion (Figure 4).

### Dilution test, pH, drug content, viscosity and Centrifugation

Dilution with water showed no phase separation indicating that optimized batch of emulsion was o/w type. pH of emulsion was 7.1, drug content 95.82 ± 1.70% and average viscosity of emulsion was found to 2000 cP. After centrifugation, no phase separation was observed which indicates that emulsion was stable.

### Evaluation of emulgel

Emulgels were found to be yellowish, white viscous creamy having good appearance, spreadability and viscosity.

### pH, Viscosity and Drug content

pH of the skin is in the range of 5.5 to 7. pH was found in range of 6.10 to 6.93 for all formulations. This indicated the compatibility of formulations to skin pH and it can show good topical delivery. Batch F6 and F7 showed higher viscosity. As the concentration of carbopol 940 increases, the viscosity of formulation was also increased. Batch F2 showed low viscosity because of low concentration of carbopol 940. The viscosity of the emulgels indicated the shear-thinning property, because as rotating speed increases, the viscosity of emulgels decreases. Batch F1 (100.90±0.37), F4 (97.45±0.61) and F7 (95.10±0.82) showed higher drug content. The drug content was within the range of 70-100%. The results indicated that the drug dispensed uniformly throughout the emulgel.

### Bio-adhesive strength measurement

Bio-adhesive strength was determined in terms of detachment stress i.e. force required to detach the formulation from membrane. Results indicated that the change in concentration of Carbopol 940 and HPMC K100M showed changes in bio-adhesive strength. The gradual increase was observed in bio-adhesive strength as the level of Carbopol 940 increased, due to availability of carboxyl group. Carbopol has very high percentage of (58-68 %) of carboxyl group that undergo hydrogen bonding with sugar residues in oligosaccharide chain in membrane. Batch F4 and F7 were shows higher bio-adhesive strength 2525.36 ± 0.13dynes/cm^2^ and 2703.06 ± 0.1213dynes/cm^2^ respectively.

### Spreadability

Spreadability of emulgel is an important parameter. With decrease in viscosity spreadability increases. Batch F1 and F4 showed highest spreadability 28.47±1.24 and 27.6±1.24 respectively. It was easily spreadable because of low viscosity. Marketed formulation showed good spreadability compare to formulated batches.

### In-vitro drug diffusion study

### Cellophane membrane 

**Figure 5:**
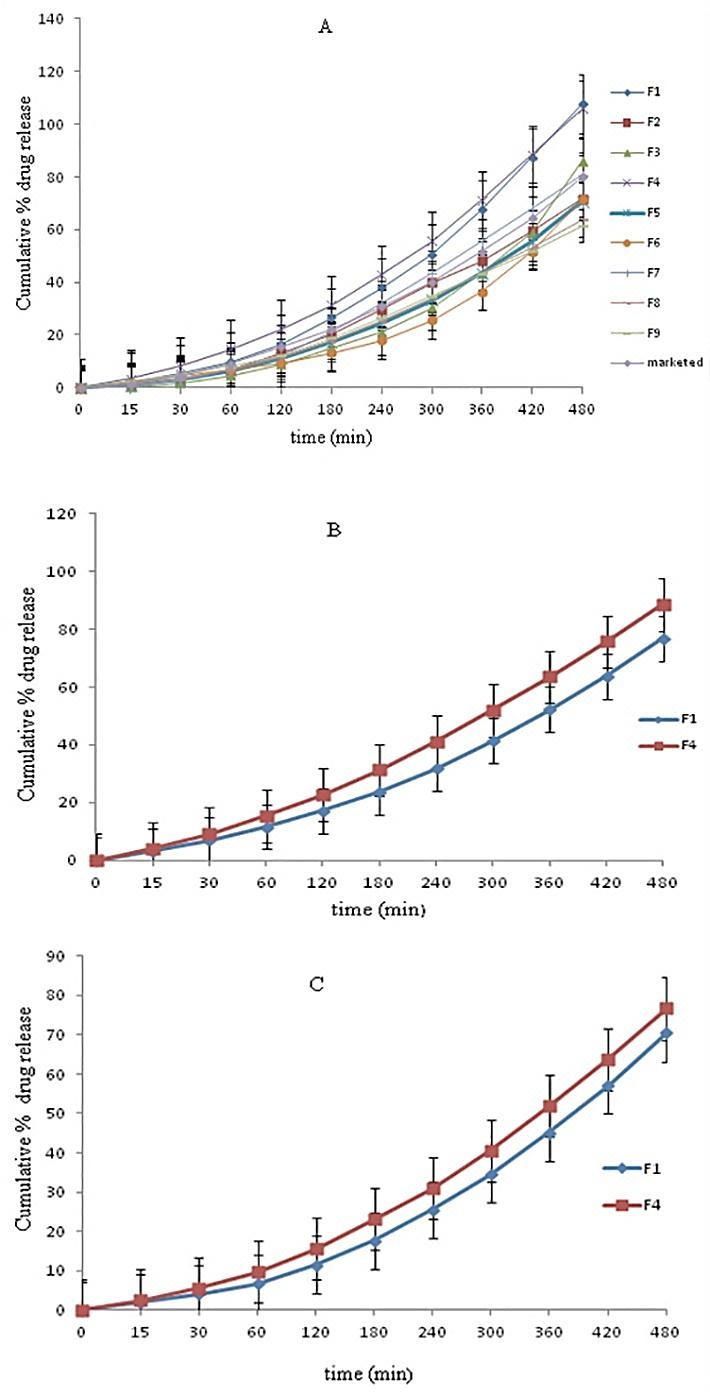
*In-vitro* Cumulative % drug release through A) Cellophane membrane for F1-F9 B) Egg membrane C)Goat skin

As shown in Figure 5A, it was found that formulation batch F1 showed 107.71 ± 2.74% and F4 showed 105.91 ± 2.74% release of drug that was faster than the other formulation due to the lower concentration of HPMC K100M and higher concentration of Carbopol 940. As there is increase in concentration of carbopol 940 which leads to increase in viscosity of the formulation and therefore decreases the drug release,^19^ Batch F9 showed 61.55 ± 2.74% was retard the drug release because the higher concentration of carbopol 940.

### Egg membrane

From the diffusion study carried out with cellophane membrane and viscosity study, batch F1 and F4 were selected for further study. At the 15 min and 30 min formulations showed less drug release compare to release through cellophane membrane (Figure 5B) because of thickness of the egg membrane. After 1hr formulations showed increase in drug release compare to release through cellophane membrane. Formulations showed less drug release after 8 hrs compare to cellophane membrane. This may be due to complexity of the egg membrane.^20^

### Ex-vivo drug permeation study 

*Ex-vivo* study of F1 and F4 formulations at 15 min and 30 min showed less drug release compare to release through cellophane membrane and egg membrane (Figure 5C). After 1hr formulations showed increase in drug release but release was less after 8hrs compared to through cellophane membrane and egg membrane. This decrease in drug release may be due to the fat content and higher thickness of goat skin. F4 showed better release than F1, this may be due to the high viscosity of F1.

### Kinetic study and mechanism of drug release

The release kinetics data based on correlation coefficient (R^2^) indicated that the release of drug for batch F4, F6, F7 and F8 from emulgels followed Zero order kinetics. Batches F2, F3 and F5 followed Korsmeyer Peppas kinetics with release component (k) values < 0.5. Batch F1 followed Hixon Crowell kinetics. Batch F9 followed Matrix kinetic.

### Experimental design (ANOVA Study)

#### Effect of Formulation Variables on Drug Release at 480 min

Drug Release(at 480 min)=+144.602-9.8850A-12.530B Equation 4

Where, A: Carbopol 940 concentration, B: HPMC K100M concentration

**Figure 6:**
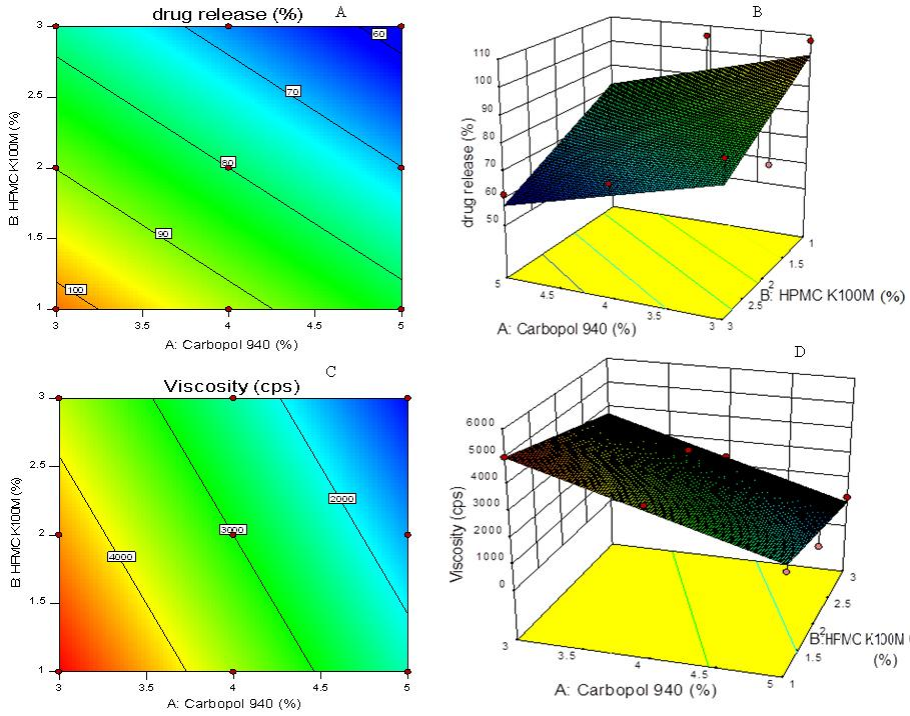
Contour plot (A, C) and response surface plot (B, D) showing relationship in between % drug release at 8 hrs and polymer concentration and viscosity of emulgel and polymers

The model terms for the drug release at 480 min. were found to be significant with high value of R^2^ 0.6634 which indicated the adequate fitting to a linear model. As it can be seen from the Figure 6A and B, the concentration of Carbopol 940 and concentration of HPMC K100M both has negative effect on release of drug (equation 4).

#### Effect of Formulation Variables on Gel Viscosity

Gel Viscosity= +9733.33+1366.667A-633.33B Equation 5

Where, A: Carbopol 940 concentration, B: HPMC K100M concentration****

The model terms for the gel viscosity was found to be significant with high value of R^2^ 0.9284 which indicates the adequate fitting to a linear model.

In design, as the concentration of carbopol 940 was increased, the gel viscosity also found to be increased. It was observed that higher concentration of carbopol 940 while lower the concentration of HPMC K100M produced a gel of higher viscosity as shown in Figure 6 C and B and equation 5.

Carbopol 940 showed greater linear effect on release of drug while HPMC K100M showed greater linear effect on viscosity of formulations as it having high degree of gelling capacity. Values of "Prob > F"(p value) less than 0.0500 indicate model terms were significant.

#### Validation of statistical model

After statistical analysis by the Design expert software, optimized batch was found to be F4. Experimental values (Table 2) for the % cumulative drug release at 480 mins and viscosity were found very close to the applied and predicted values indicating successful validation for the model.

**Table 2:** Analysis of Variance

**Sr. No.**	**Response Model**	**Sum of Squares**	**Df**	**Mean Square**	**F value**	**P value**	**R** ^ 2 ^	**Adequate Precision**
1	**Drug Release (480 min.)**	1528.28	2	764.14	5.91	0.0381	0.6634	6.830
2	**Viscosity**	1.361E+007	2	6.807E+006	18.34	0.0028	0.8594	11.373

#### Flux

Diffusion flux measures the amount of substance that will flow through a small area during a small time interval. Flux was obtained from the slope values plotted for amount diffused per unit area against time. Flux of F1-F9 and marketed formulation were determined through cellophane membrane. The flux of the formulation was in the range of 0.780-1.273 µg/cm^2^/min. The flux of batch F4 was found to be more than batch F1 and marketed formulation through cellophane membrane. Drug incorporation in emulgel base and use of PEG 400 as permeation enhancer were responsible for enhancement of flux.

#### Similarity factor 

Similarity factor (f2) was found to be 39. So, it was concluded that optimized batch was not similar to the marketed formulation.

#### Microbiological assay

The % inhibition of pure drug found to be higher than optimized batch F1, F4 and marketed (Table 3). This may be due to slow release of drug from emulgel network.

**Table 3:** Zone of inhibition

**Micro-organism**	**Formulation**	**L1 (cm)**	**L2 (cm)**	**Zone of inhibition (%)**
***Staphylococcus aureus***	F1	9	3.0	33.33±1.52
	F4	9	3.1	34.44±1.74
	Marketed	9	3.5	38.88±1.50
	Pure drug	9	4.1	45.55±1.60
***E .Coli***	F1	9	2.0	22.22±0.80
	F4	9	2.5	27.77±1.25
	Marketed	9	3.3	36.66±1.36
	Pure drug	9	4	44.44±1.85

L1 = total length of the streaked culture (cm)

#### Stability Study

From the stability for batch F4, it was observed that there was no significant change on evaluation parameters before and after the study.

## Conclusion

In conclusion, a stable, elegant and effective transdermal emulgel delivery for Ofloxacin has developed. The delivery was optimized using HPMC K100M and Carbopol 940 as a gelling agent. Emulgel exhibited good *in-vitro* drug release and viscosity. Emulgel will act as depot of drug which will release drug in controlled manner at the targeted site. Hence the optimized formulation F4 may be used to treat the topical bacterial diseases.

## Acknowledgments

Authors are sincerely thankful to Mercury Laboratories Pvt. Ltd. for providing gift sample of drug. Authors would like to thank Colorcon Asia Pvt. Ltd., Mumbai, India for providing gift sample of HPMC. Authors are highly grateful to Dr. B. S. Kuchekar, Principal and management of MAEER’S Maharashtra Institute of Pharmacy, Pune, India for moral support and providing necessary infrastructure to carry out research work.

## Ethical Issues

Not applicable.

## Conflict of Interest

The authors declare no conflict of interests.
